# A hindbrain inhibitory microcircuit mediates vagally-coordinated glucose regulation

**DOI:** 10.1038/s41598-019-39490-x

**Published:** 2019-02-25

**Authors:** Carie R. Boychuk, Katalin Cs. Smith, Laura E. Peterson, Jeffery A. Boychuk, Corwin R. Butler, Isabel D. Derera, John J. McCarthy, Bret N. Smith

**Affiliations:** 1 Department of Physiology, College of Medicine, University of Kentucky, Lexington, KY USA; 2 Department of Neuroscience, College of Medicine, University of Kentucky, Lexington, KY USA

## Abstract

Neurons in the brainstem dorsal vagal complex integrate neural and humoral signals to coordinate autonomic output to viscera that regulate a variety of physiological functions, but how this circuitry regulates metabolism is murky. We tested the hypothesis that premotor, GABAergic neurons in the nucleus tractus solitarius (NTS) form a hindbrain micro-circuit with preganglionic parasympathetic motorneurons of the dorsal motor nucleus of the vagus (DMV) that is capable of modulating systemic blood glucose concentration. *In vitro*, neuronal activation or inhibition using either excitatory or inhibitory designer receptor exclusively activated by designer drugs (DREADDs) constructs expressed in GABAergic NTS neurons increased or decreased, respectively, action potential firing of GABAergic NTS neurons and downstream synaptic inhibition of the DMV. *In vivo*, DREADD-mediated activation of GABAergic NTS neurons increased systemic blood glucose concentration, whereas DREADD-mediated silencing of these neurons was without effect. The DREADD-induced hyperglycemia was abolished by blocking peripheral muscarinic receptors, consistent with the hypothesis that altered parasympathetic drive mediated the response. This effect was paralleled by elevated serum glucagon and hepatic phosphoenolpyruvate carboxykinase 1 (PEPCK1) expression, without affecting insulin levels or muscle metabolism. Activity in a hindbrain inhibitory microcircuit is sufficient to modulate systemic glucose concentration, independent of insulin secretion or utilization.

## Introduction

The brain orchestrates peripheral responses to changes in blood glucose concentration^1–3^. Several recent studies have identified insulin-independent pathways for regulating glucose metabolism^4–9^, and the role of the brain as a glucose regulatory organ^10^ is gaining acceptance. Multiple ‘preautonomic’ areas of the brain contribute to systemic glucose homeostasis^11–13^. Much work describing the glucose regulatory function of the brain focuses on the hypothalamic microcircuitry necessary for feeding and satiety^6,14–17^, but ample evidence indicates that the brainstem dorsal vagal complex (DVC) plays an important role in modulating plasma glucose and insulin levels, feeding, and energy balance^18–22^. Vagally-mediated parasympathetic output critically regulates visceral functions related to metabolic homeostasis^9,23^. The cephalic phase of insulin release requires an intact vagus nerve^24^ and lesioning the hepatic vagus nerve potently suppresses effects of central insulin application on hepatic gluconeogenesis in diabetic mice^25^. Moreover, injection of a glucoprivic glucose analogue into the vagal complex, but not hypothalamic areas, increases both feeding and hyperglycemia in rats^18^. Neurons in the DVC clearly influence blood glucose concentration, yet little is known about the circuitry underlying this effect.

Vagal motor output to the thoracic and most abdominal viscera is generated by the preganglionic, parasympathetic motor neurons of the dorsal motor nucleus of the vagus (DMV), making the DMV the final, central modulatory point in parasympathetic activity. DMV motor neurons are tonically active, but this activity is modulated on a moment-to-moment basis by synaptic inputs from second order viscerosensory neurons in the nucleus tractus solitarius (NTS); together with the area postrema, these structures comprise the DVC. Microinjection of nutrient signaling molecules into the DVC alters overall blood glucose concentration^18,26,27^ and vagal activity^28^. Relatively few vagal motor neurons are considered glucose-sensitive^29^, and the ability to respond to metabolic changes is likely communicated via synaptic transmission from other nuclei. Neurons throughout the DVC are directly responsive to a wide range of nutrient and satiety signals, including leptin^30,31^, insulin^26,32,33^, lactate^34^, and glucose^18,29,35–39^. In particular, most inhibitory GABAergic neurons within the NTS receive primary vagal afferent input^40,41^ and are themselves glucose responsive^35,36^, and GABAergic NTS neurons project prominently to the DMV^42^ contributing significantly to vagal motor neuron activity^43,44^. GABA release in the DMV is also significantly elevated after the induction of diabetes^45^, consistent with diabetes-induced plasticity of DMV neuronal function^46,47^, particularly of GABA_A_ receptor activity^48,49^. Thus, GABA neurons in the NTS are positioned to potently modulate vagal motor activity in response to neural and circulating metabolic signals, but their influence on systemic glucose regulation is unknown.

Taken together, the DVC, particularly its GABAergic circuitry, appears ideally situated to regulate peripheral glucose metabolism. However, this function remains controversial. Electrical stimulation of the vagus nerve causes the release of both insulin and glucagon from the pancreas^50–54^. However, the release of these hormones with opposite effects on systemic glucose levels makes it difficult to predict how vagal activity changes modulate blood glucose concentration. Manipulating activity of glucose sensing neurons in the NTS results in changes in gastric motility^28^ and glucagon secretion^36^, but neither of these assessments probed whether direct modulation of inhibitory neurocircuitry in the DVC alters blood glucose concentration. We hypothesized that experimentally increasing the activity of GABAergic neurons in the DVC would inhibit DMV motor neurons and increase blood glucose concentration, whereas experimentally decreasing the activity of GABAergic neurons in this region would have the opposite effects.

## Results

**Figure 1:**
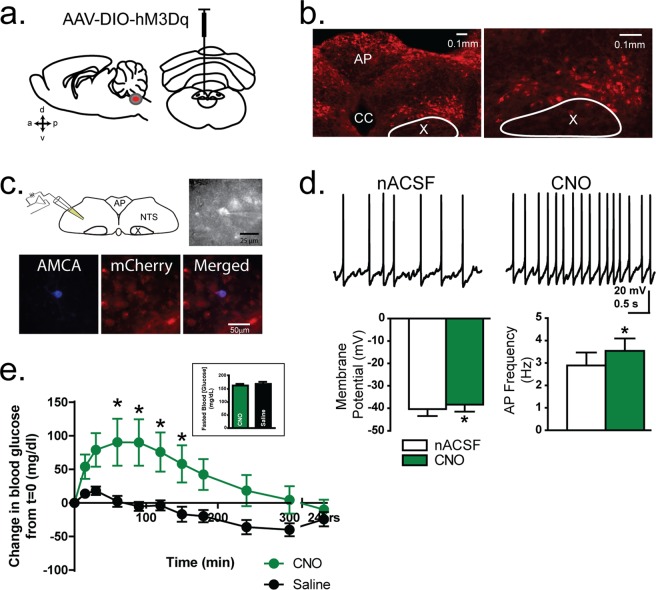
Cre-dependent expression and response to activation of designer receptor exclusively activated by designer drugs (DREADDs) in dorsal hindbrain GABAergic neurons. (**a**) Expression of excitatory DREADDs was induced though a stereotaxic microinjection of pAAV8-hSyn-DIO-hM3Dq into the dorsal vagal complex (DVC) of a vGAT-Cre mouse. (**b**) Light microscopic images of endogenous mCherry expression in the DVC. Left: low magnification image of the DVC. Right: higher magnification centered on the nucleus of the solitary tract (NTS) three weeks after AAV inoculation. The dorsal motor nucleus of the vagus (X) is encircled. Expression was prominent in the NTS, but also could include a few GABAergic interneurons located in the DMV, which have been described previously^56^. (**c**) Illustration of the recording configuration, with the recording pipette in the NTS. Image under combined fluorescent and IR/DIC illumination during a recording from an mCherry expressing NTS neuron is to the right of the diagram. Recording pipette is visible attached to an mCherry expressing neuron. Lower. Images of an NTS neuron that was filled with biocytin during a recording and was visualized post hoc with AMCA (left); the same section using a filter to visualize mCherry (middle); and merged images (right) demonstrating that the recorded neuron expressed mCherry. (**d**) Top: Action potentials in an mCherry expressing NTS neuron recorded under control conditions (nACSF) and in the presence of clozapine-N-oxide (CNO; 10 µM) Bottom: Mean (±SEM) membrane potential (mV) and action potential firing rate (Hz) were both significantly affected by CNO application (n = 8; p < 0.05). (**e**) Blood glucose concentration after intraperitoneal (i.p.) administration of CNO (1 mg/kg) or vehicle (0.9% saline + 0.5% DMSO). Asterisks indicate significant effect of CNO (p < 0.05). Inset: Graph of mean fasted blood glucose concentration *in vivo* before CNO or vehicle administration. No differences were detected prior to treatment.

Traditionally, manipulations of neurotransmission in the DVC were accomplished using electrical, photochemical, or pharmacological stimulation methods^19,50,55^, but these techniques do not provide the phenotype selectivity needed to target the GABAergic neuron sub-population in the dorsal hindbrain. We stimulated GABAergic NTS neurons selectively by utilizing a chemogenetic approach, using a stereotaxically injected cre-recombinase-inducible adeno-associated viruses expressing the chemogenetic designer receptor exclusively activated by designer drugs (DREADDs) and the fluorescent marker, mCherry, into the DVC of mice that express cre-recombinase in GABAergic neurons (i.e., vGAT-Cre mice, Slc32a1tm2(cre)lowl/J; the Jackson Laboratories; stock 016962; Fig. 1a).

### Activation of GABAergic Hindbrain Neurons Using hM3Dq-mCherry

Widespread mCherry+ expression was present throughout the NTS in the dorsal hindbrain 3–4 weeks after viral transduction, the general distribution of which matched that observed for GABAergic NTS neurons reported previously^41,56,57^ (Fig. 1b). We initially tested whether GABAergic NTS neurons expressing hM3Dq-mCherry (AAV8 DIO hM3Dq) increased their activity in response to the DREADD ligand, clozapine-N-oxide (CNO; 10 µM). mCherry-expressing NTS neurons were targeted for patch-clamp recordings in acutely prepared brainstem slices (Fig. 1c). A modest, but consistent and significant depolarization was observed in mCherry-expressing neurons exposed to CNO (ΔmV = +2.0 ± 0.2 mV; n = 7; p = 0.00002 paired t-test; Fig. 1d). This depolarization was accompanied by a significant increase in action potential firing (2.9 ± 0.6 Hz in normal artificial cerebrospinal fluid (nACSF) versus 3.5 ± 0.5 Hz in CNO; p = 0.002; n = 8). After a 15 minute washout, action potential firing was similar to that prior to drug treatment (p = 0.2 vs pre-drug application). Application of CNO had no effect on either membrane potential (p > 0.05) or action potential frequency (p > 0.05) in any unlabeled, non-transfected NTS neuron (n = 4). A randomly-selected subset of recorded mCherry+ neurons expressed GAD67 mRNA, determined by single-cell RT-PCR^35^, confirming their GABAergic phenotype (n = 6; data not shown).

### Activation of Hindbrain GABAergic Circuits Increases Blood Glucose Concentration

We employed a counter-balanced experimental design, where each animal served as its own control, wherein 50% of the animals received one treatment (i.e. vehicle versus CNO) while the other half received the other treatment on any day of testing, to investigate how activation of GABAergic neurons in the dorsal hindbrain affects blood glucose concentration. After a two hour fast, baseline blood glucose levels were not different between groups before receiving saline (169.5 ± 7.6 mg/dL) or CNO injection (162.9 ± 6.6 mg/dL; n = 7; p = 0.5; Fig. 1e). After systemic CNO (1 mg/kg) intraperitoneal administration, blood glucose concentration rose steadily compared to an injection of the vehicle (0.9% NaCl + 0.5% DMSO). This rise was apparent within 15 min and became significant at 60–90 minutes (Repeated Measures ANOVA with Tukey’s post-hoc test; interaction p = 0.0002; Fig. 1e). These data provide direct evidence that increased activity of GABAergic, DVC neurons increases peripheral blood glucose concentration.

A recent study suggested that CNO may have off-target effects due to potential actions of its metabolites^58^. Additional control experiments were performed to test the effect of CNO in animals with no detectable mCherry expression in the DVC (e.g. injections that missed the DVC), or animals that did not receive hM3Dq-mCherry virus. Counterbalanced intraperitoneal injections of saline or CNO had no effect on blood glucose concentration in any of these controls (p = 0.7; n = 7). A significant reduction in blood glucose was observed over time as a result of fasting conditions in all mice (Repeated Measures ANOVA; time p < 0.0001; Supplemental Fig. S1). However, there was no difference in the effect on blood glucose concentration of vehicle versus CNO administration in these mice (Repeated Measures ANOVA; interaction p = 0.3; CNO versus saline p = 0.2; Supplemental Fig. S1). Therefore, the ability of CNO to increase systemic blood glucose required the activation of hM3Dq-mCherry expressing GABAergic neurons in the DVC.

### Activation of Hindbrain GABAergic Circuits Inhibits Vagal Motor Activity

**Figure 2:**
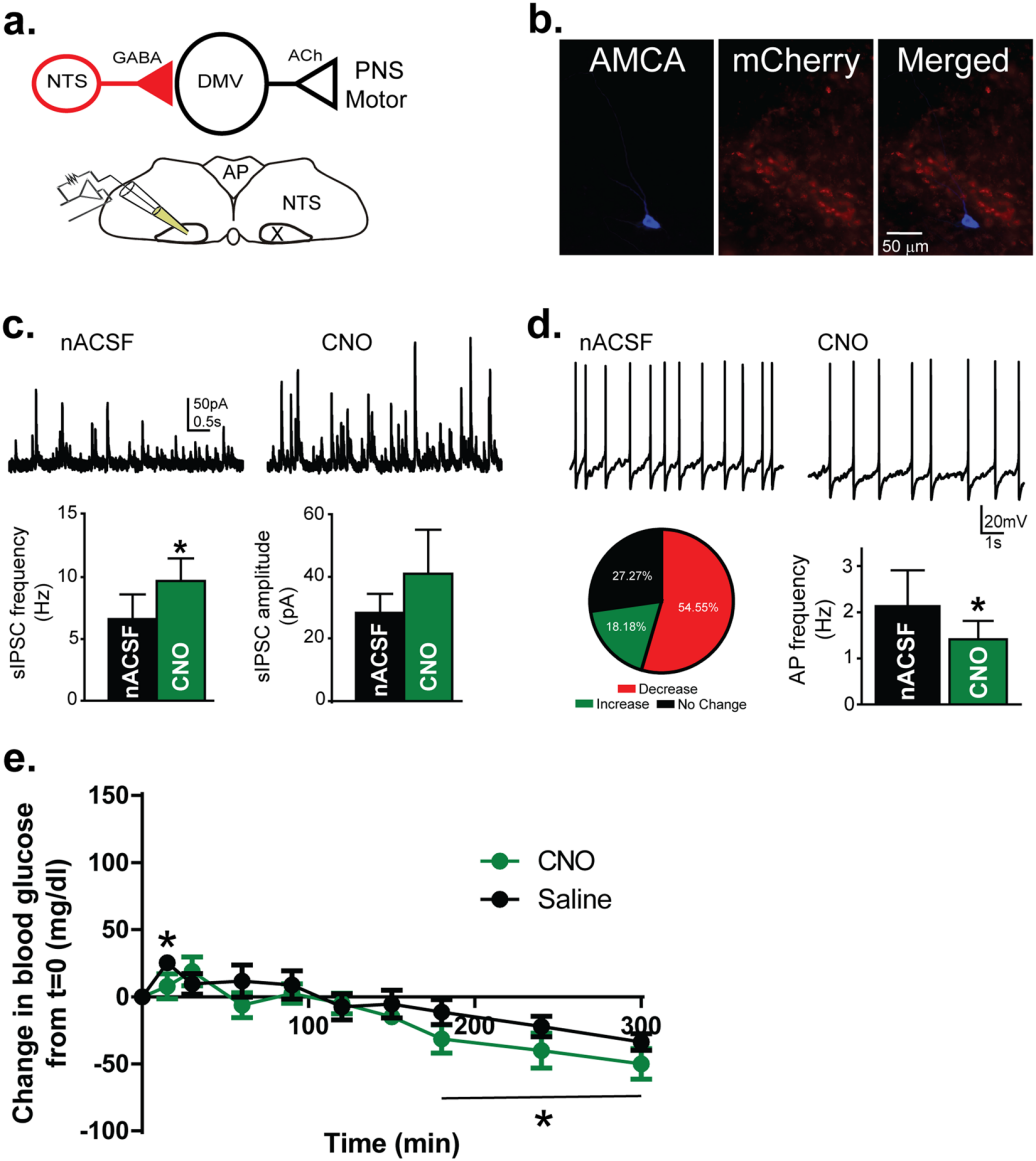
Activation of GABAergic neurons in the DVC inhibits activity of neurons in the dorsal motor nucleus of the vagus (DMV). (**a**) Top: Illustration of the synaptic pathway tested. A GABA neuron in the NTS with a synaptic connection to a motor neuron in the DMV (X) is illustrated. Bottom: Illustration indicating the location of recordings in the DMV. (**b**) Post hoc confirmation of the location of a recorded and biocytin-filled DMV neuron. No co-localization with mCherry was observed in DMV neurons. (**c**) Top: Spontaneous inhibitory postsynaptic currents (sIPSCs) in a DMV neuron before and after CNO application. Bottom: Mean sIPSC frequency and amplitude (n = 6). CNO significantly increased sIPSC frequency (p < 0.05), but not amplitude. (**d**) Top: Action potential firing in a DMV neuron before and after CNO application. Bottom: Pie graph illustrating the proportion of AP firing responses in DMV neurons to bath application of CNO (left) and mean action potential (AP) frequency in the neurons in which a decrease in AP frequency was induced by CNO (n = 5; p < 0.05; right). (**e**) Mean blood glucose concentration after *in vivo* systemic administration of CNO (1 mg/kg) or vehicle (0.9% saline + 0.5% DMSO) in mice pre-treated with muscarinic receptor antagonist, methylscopolamine (1 mg/kg; i.p.). There was no effect of CNO administration in the presence of MSA. Asterisks indicate significant differences from time 0, but no differences between treatment groups.

Inhibitory neurons in the NTS send significant, functional projections to preganglionic DMV motor neurons^42,44,59^, whose axons course via the vagus nerve to postganglionic neurons located in organs important for glucose metabolism^60–62^. Assuming the glucose-altering effects of GABAergic NTS neuron activity was mediated by vagal projections, we hypothesized that the chemogenetic activation of inhibitory neurons in the DVC would dampen excitability of DMV motor neurons. Using whole-cell voltage-clamp recordings from brainstem slices, we assessed whether hM3Dq-mediated activation of inhibitory NTS neurons affected inhibitory synaptic signaling to DMV motor neurons. Application of CNO resulted in a significant increase in the frequency of inhibitory postsynaptic currents (IPSCs) in DMV motor neurons (9.3 ± 2.5 Hz in nACSF versus 12.2 ± 2.4 Hz in CNO; p = 0.04; n = 8; Fig. 2c). The mean IPSC amplitude was not reliably changed by CNO (p = 0.2). These data indicate that hM3Dq-mediated activation of GABAergic NTS neurons increases functional synaptic inhibition of the DMV.

Although pharmacological approaches have shown that GABAergic neurotransmission robustly modulates DMV motor neuron activity^43,44^, we further investigated if the hM3Dq-induced increase in inhibitory neurotransmission to the DMV could alter motor neuron activity. Consistent with the increase in IPSC frequency, the majority of DMV neurons exhibited a decrease in action potential firing after CNO application (≥15% change: 6 decreased; 3 no change; 2 increased; n = 11; Fig. 2d). The decrease in action firing was accompanied by a hyperpolarization of the resting membrane potential of 3.4 ± 0.9 mV. This experiment was repeated in the presence of the type A GABA receptor (GABA_A_) blocker, bicuculline methiodide (BIC; 30 µM; n = 4). In the presence of bicuculline, CNO failed to decrease action potential firing in any neuron recorded, confirming that GABAergic neurotransmission mediated the decrease in DMV neuron activity after CNO administration. These results indicated that activation of GABAergic neurons in the DVC inhibits activity of vagal motor neurons and that the hM3Dq DREADDs approach used here produced the anticipated cellular effects on the DVC.

### Modulation of Blood Glucose Concentration is Vagally-Mediated

Acetylcholine (ACh) released from postganglionic parasympathetic neurons binds muscarinic receptors (mAChRs) in peripheral target cells, and mAChR antagonists selectively block parasympathetic nervous system functions^63^. If activation of GABAergic neurons in the DVC works through vagal parasympathetic pathways, we reasoned that blocking mAChRs peripherally would mitigate the effect of systemic CNO administration on blood glucose concentration. To test this hypothesis, mice were systemically pre-treated with a mAChR antagonist that does not cross the blood-brain barrier, (−)-scopolamine methyl bromide (i.e., methylscopolomine; MSA; 1 mg/kg). Administration of MSA alone significantly increased blood glucose concentration (Supplemental Fig. S2). Using a counter-balanced experimental design, the effect of CNO on blood glucose concentration was abolished when mAChRs were blocked by MSA pre-administration (Repeated Measure ANOVA; p = 0.2; Fig. 2e). Taken together, these results demonstrate that activation of hindbrain inhibitory circuits increases blood glucose via a GABA_A_- and mACh receptor-dependent, vagally-mediated signaling pathway.

### Peripheral Pathways Mediating CNO-Induced Hyperglycemia

**Figure 3:**
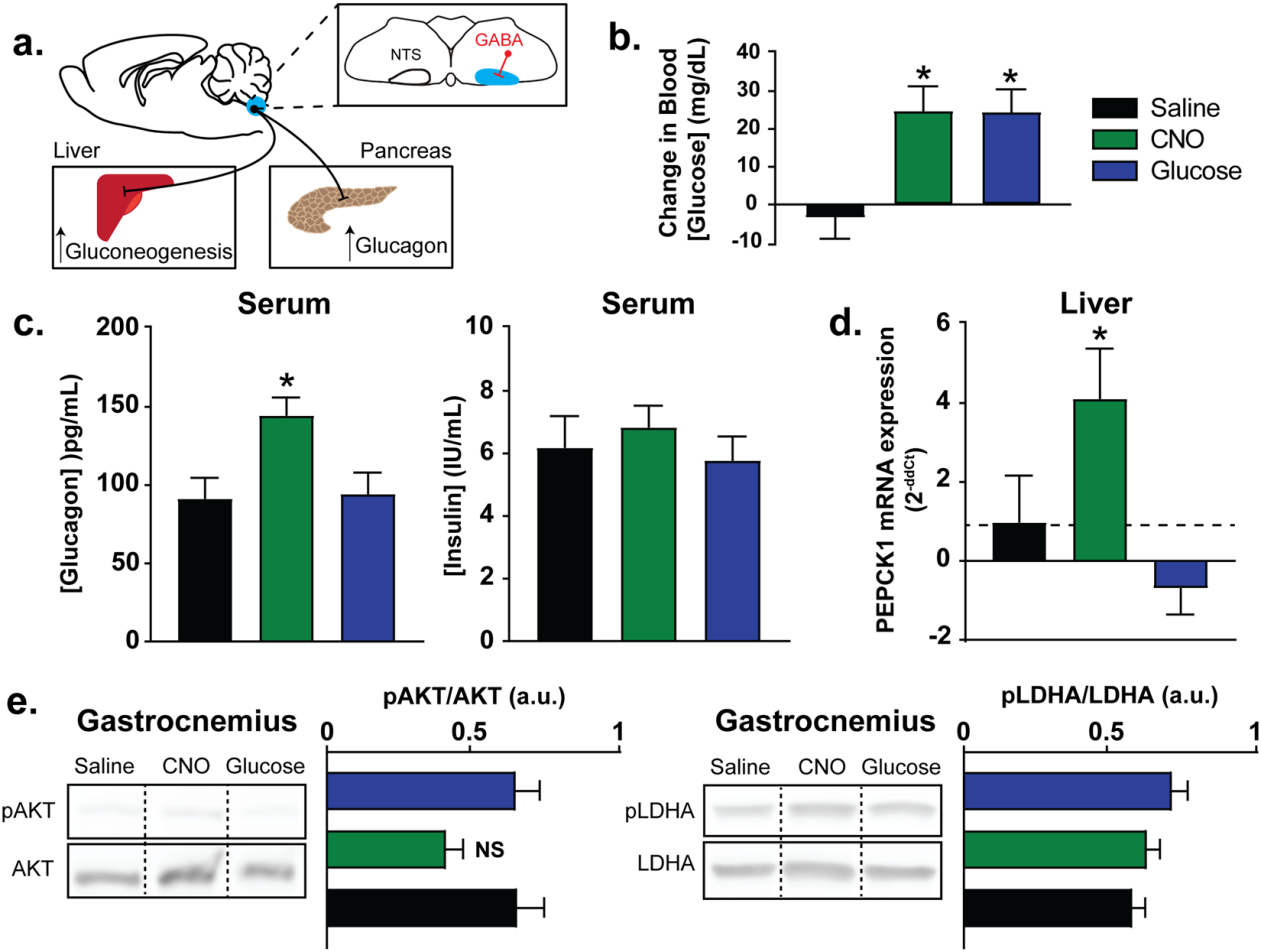
Peripheral pathway mediating CNO-induced hyperglycemia. (**a**) Illustration of hypothesized pathway involved in the elevation of blood glucose after remote activation of GABAergic hindbrain neurons. The inhibitory synaptic connection from NTS neurons to the DMV is shown, as is the increase in glucagon release from the pancreas. Increased hepatic gluconeogenesis could result from decreased vagal input (shown) and/or glucagon actions in the liver. (**b**) Change in blood glucose after 90 mins of vehicle, CNO (1 mg/kg), or glucose (0.26 mg/kg) injection (i.p.). (**c**) Mean serum glucagon (left) and insulin (right) concentration 90 min post-treatment. (**d**) Mean expression (fold change) of hepatic PEPCK1 mRNA expression (normalized to β-actin expression). (**e**) Western blots indicated that pAKT/AKT and pLDHA/LDHA protein expression in gastrocnemius muscle were unaltered at the same time point. For each protein, example blots were cropped from the same gel, with boxes and lines added for clarity; exposures are unaltered. The full-length blots are shown in Supplemental Fig. S4.

To examine the potential peripheral targets mediating DREADD-induced elevation in blood glucose concentration, vGAT-Cre mice injected with the hM3Dq construct in the dorsal hindbrain mice were injected (i.p.) with either CNO, saline vehicle, or a bolus of glucose (0.26 mg/kg) to mimic the elevation in the blood glucose concentration that was observed after CNO injection. Mice were sacrificed after 90 mins and various tissues were assessed for their potential role in mediating the elevation in blood glucose concentration. Previous reports using optogenetic activation of hindbrain glucose transporter 2 (Glut2)-expressing neurons suggested that vagal circuits can modulate pancreatic secretions^36^. Therefore, we tested plasma samples for markers of pancreatic hormone release. Consistent with that previous report, CNO-mediated activation of hindbrain GABAergic neurons elevated serum glucagon levels *in vivo* (p = 0.01; n = 6; Fig. 3c). Of particular note, serum insulin concentration did not change (p = 0.66; n = 6; Fig. 3c). Importantly, no differences existed between the vehicle-injected animals and those receiving a bolus of glucose 91.87 ± 12.9 versus 94.99 ± 13.11 IU/mL (Fig. 3c), indicating that the effect of CNO on glucagon concentration was not simply in response to elevated glucose levels. These data demonstrate that activation of inhibitory signaling in the dorsal hindbrain increases glucagon release, and extend previous reports by demonstrating that this is specific to glucagon release, since insulin concentration was not changed.

Since changes in insulin sensitivity can profoundly affect blood glucose concentration independently of changes in total serum insulin levels, we tested whether peripheral insulin sensitivity was altered in skeletal muscle after GABAergic dorsal hindbrain activity. To accomplish this, we assessed AKT activation in gastrocnemius muscle after DREADDs-mediated activation of GABA neurons in the DVC. Consistent with the lack of change in serum insulin levels, there was no detectable change in the basal levels of Akt (ANOVA; p = 0.62), the phosphorylated state of Akt (pAKT; ANOVA; p = 0.31) or the ratio of pAKT/AKT (ANOVA, p = 0.07; Fig. 3e) 90 min after CNO injection. A previous report demonstrated that brain-mediated remission of hyperglycemia involves changes in lactate and pyruvate production in muscle tissue^7^. Therefore, we also examined lactate dehydrogenase A activity (LDHA). There was a significantly lower level of LDHA in CNO-treated animals compared to both vehicle- (ANOVA; p = 0.0004) and glucose-injected mice (p = 0.002) (Supplemental Fig. S3), consistent with decreased lactate production in the muscle tissue. There was also a significantly lower level of activated/phosphorylated state LDHA (pLDHA) in the CNO-treated group versus glucose injected mice (ANOVA; p = 0.0073), but neither of these groups were different from vehicle injected mice. Despite these difference in absolute LDHA content, there was no significant difference in the ratio of pLDHA/LDHA across any of the groups (ANOVA, p = 0.15) suggesting no consistent treatment related change occurred.

Previous reports suggested that hindbrain circuitry modulates blood glucose concentration through changes in hepatic gluconeogenesis^5,9^. Similarly, we found a significant upregulation of hepatic phosphoenolpyruvate caroboxykinase 1 (PEPCK1) expression in animals administered CNO (2^−ddCt^: 4.1 ± 1.3 fold; n = 10; p = 0.02; Fig. 3d) compared to vehicle (n = 10). In mice receiving a single bolus of glucose, PEPCK1 expression (2^−ddCt^: −0.7 ± 0.6; n = 7) was slightly lower than in vehicle-injected mice (≤20%) 90 min post-injection, but this was not statistically significant (Fig. 3d; p = 0.6).

### Decreasing Activity of GABAergic Hindbrain Neurons Using hM4Di-mCherry

**Figure 4:**
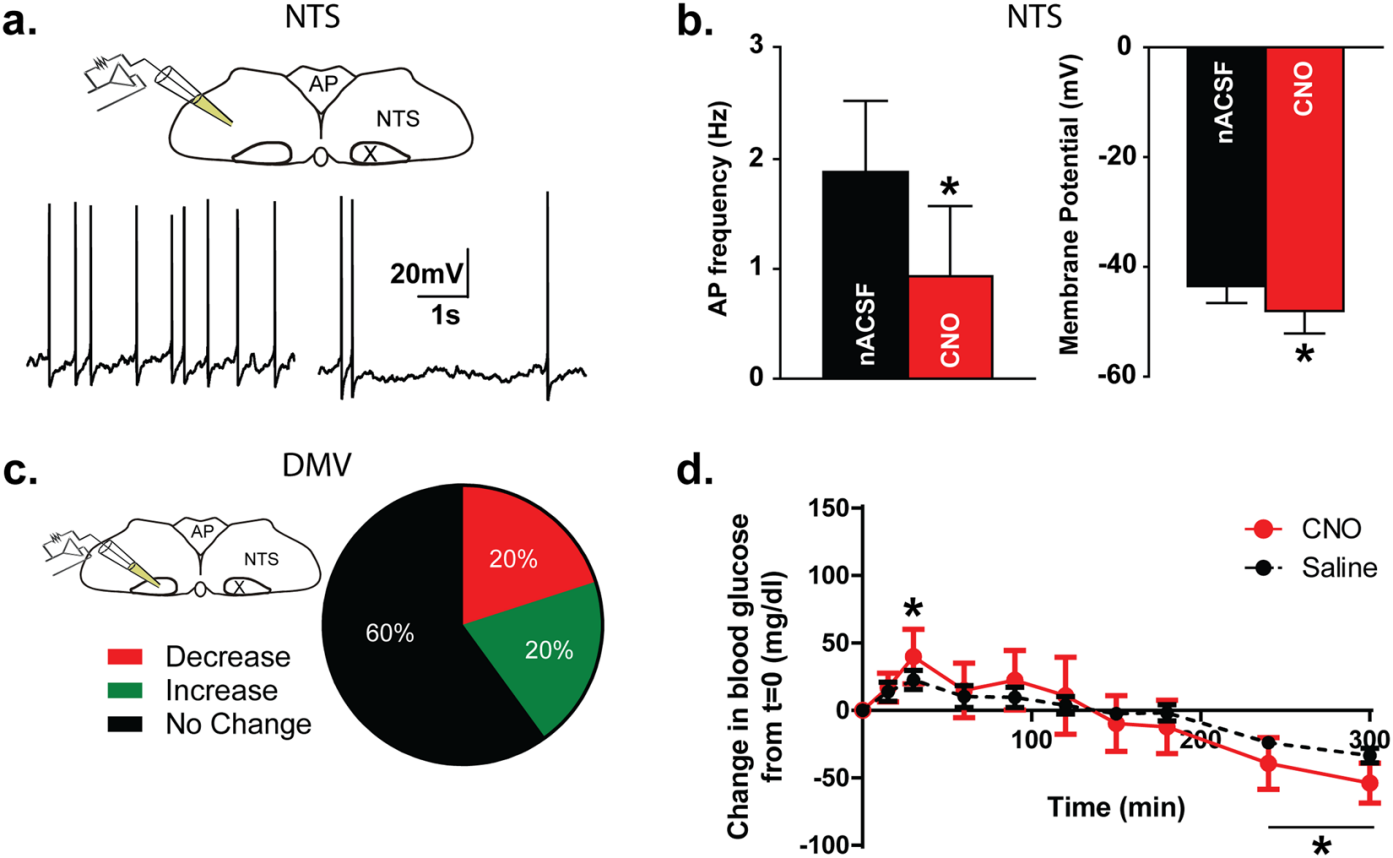
Activation of the inhibitory hM4Di DREADD in GABAergic hindbrain neurons does not alter blood glucose concentration or DMV neuron activity. (**a**) Diagram showing recording location in the NTS and representative traces showing action potentials in an mCherry expressing NTS neuron before (left) and after CNO (right; 10 μM) application in a mouse that received the hM4Di viral construct. (**b**) Mean AP firing and membrane potential before and after CNO application (n = 8). *Significant difference from nACSF (paired t-test; p < 0.05). (**c**) Diagram showing recording location in the DMV (left). Pie graph (right) illustrating AP firing responses in NTS and DMV neurons. AP firing was decreased by CNO in NTS neurons but was unchanged in most DMV neurons (n = 5). (**d**) Blood glucose concentration after *in vivo* administration of CNO (1 mg/kg) or vehicle (0.9% saline + 0.5% DMSO). Asterisks indicate significant change from time 0, but no differences between treatment groups.

Using the same stereotaxic approach as previously described for hM3Dq, we virally transduced a separate cohort of vGAT-Cre mice with AAV8-DIO-hM4Di-mCherry, the inhibitory form of the DREADDs. Recordings from mCherry-labeled NTS neurons indicated a significant hyperpolarization after exposure to CNO (ΔmV = −4.6 ± 1.2 mV; n = 5; 10 µM CNO; p = 0.008 paired t-test; Fig. 4a,b). This hyperpolarization was accompanied by a significant decrease in action potential firing (2.0 ± 0.6 Hz, nACSF versus 1.2 ± 0.6 Hz, CNO; p = 0.002; n = 8). Action potential firing returned to baseline after a 15 minute washout to nACSF (p = 0.3). Application of CNO reduced activity of mCherry-expressing NTS neurons, consistent with the expression of the inhibitory DREADD construct in these neurons.

### Inhibiting GABAergic Hindbrain Neurons does not Modulate Blood Glucose Concentration or Vagal Motor Neuron Activity

The same counterbalanced experimental design as used for hM3Dq was employed to test the effect of activating hM4Di in GABAergic hindbrain neurons. In this case, no change in blood glucose concentration was detected after CNO injection in vGAT-Cre mice despite hM4Di’s significant dampening of activity of NTS circuitry *in vitro* (Fig. 4d). Therefore, despite robust glucose changes after GABAergic NTS neuron activation using hM3Dq, dampening of activity in these same neurons with hM4Di did not evoke changes in blood glucose concentration.

Based on the lack of effect on whole animal blood glucose concentration, we then tested whether DREADDs-induced suppression of GABAergic hindbrain neuron activity affected GABAergic signaling to downstream DMV motor neurons. Application of CNO significantly decreased the frequency of IPSCs in recorded DMV motor neurons (7.5 ± 2.2 Hz in nACSF versus 5.1 ± 1.6 Hz in CNO; p = 0.05; n = 7). Unlike the hM3Dq, however, the amplitude of inhibitory events was also altered after hM4Di-dependent inhibition of GABAergic hindbrain neurons, being reduced by ~10% (30.0 ± 2.3 pA in nACSF versus 26.9 ± 2.7 pA in CNO; p = 0.04; n = 7). The effect of hM4Di activation on IPSC amplitude may be due to a reduction of synchronous release from pre-synaptic terminals. Therefore, we concluded that hM4Di-expressing inhibitory NTS neurons indeed project to the DMV and reducing their activity with CNO resulted in decreased GABA release in the DMV.

We then tested whether this dampened neurotransmission translated into disinhibition and increased action potential firing of DMV motor neurons. Unlike CNO-mediated activation of GABAergic circuits, inhibition of hM4Di-expressing GABA neurons with CNO did not produce consistent changes in DMV action potential firing (≥15% change: 1 decrease; 1 increase; 3 no change; n = 5; Fig. 4c). Although inhibitory synaptic input to the DMV was decreased by activation of hM4Di in GABAergic neurons, DMV motor neuron activity was unaltered overall. This lack of effect on action potential firing in DMV motor neurons is consistent with the lack of CNO-evoked changes in systemic blood glucose concentration.

## Discussion

Despite our appreciation of fundamental autonomic pathways and principles, the role of parasympathetic output in metabolic regulation, including maintenance of blood glucose concentration, is not well understood. Given the importance of GABAergic inhibition in the modulation of parasympathetic drive to the subdiaphragmatic viscera^28,43,44,64,65^, we investigated how dorsal hindbrain inhibitory neurons contribute to systemic blood glucose regulation, using a chemogenetic approach to remotely activate/deactivate GABAergic inhibition within the dorsal hindbrain. Effects of DREADDs activation were investigated at the cellular level using patch-clamp recordings in the NTS and DMV and cellular activity was subsequently linked to whole animal glucose metabolism. Results here demonstrate that remote chemogenetic activation of GABAergic NTS neurons is sufficient to elevate blood glucose concentration. Blocking mAChRs with MSA increased blood glucose concentration and prevented the CNO-induced increase in glucose concentration, consistent with a vagally-mediated change in peripheral glucose metabolism. The effect on blood glucose was paralleled by increased glucagon release in the absence of a change in serum insulin concentration or insulin sensitivity; hepatic PEPCK1 expression was also increased, suggesting that CNO also increased gluconeogenesis in the liver. The effects of CNO-dependent activation of hM3Dq-expressing GABAergic NTS neurons on glucagon secretion and hepatic gluconeogenesis are consistent with a reduction in vagally-mediated parasympathetic output to the pancreas and liver; the effect on hepatic PEPCK1 expression could also be secondary to hormonal effects of elevated glucagon. Taken together, these results provide evidence of a dorsal hindbrain mechanism for rapidly induced hyperglycemia, independent of insulin.

Recent studies have suggested that CNO-activated DREADD systems may not have the specificity initially assumed due to possible effects of CNO metabolites, including clozapine^58^, but results here are consistent with DREADDs-specific responses. Importantly, all of the effects we observed were restricted to animals that expressed DREADDs within the DVC; animals that lacked hM3Dq DREADDs expression in GABAergic hindbrain neurons failed to exhibit any systemic or cellular effects of CNO. There were also divergent effects of the Gq and Gi forms of DREADDs, which would be unexpected if CNO or its byproducts were binding endogenously expressed receptors. Such off-target pharmacological effects would be expected to be identical, independent of the type of DREADDs expressed, and should also have occurred when no DREADDs were expressed, neither of which was the case. The *in vitro* patch-clamp studies demonstrated that DREADDs-mediated changes in either action potential firing or GABAergic neurotransmission were rapid (occurring in less than 5 mins), were consistent with the expected excitatory or inhibitory actions of DREADDs activity, and failed to detect any complex or multimodal effects of CNO that might indicate off-target effects of clozapine. The metabolic byproducts of CNO preferentially affect DREADDs, with no off-target effects reported at doses similar to those used here, and were only apparent after long exposure periods, during which accumulation of clozapine would be expected to increase its concentration^58,66^. Changes in blood glucose concentration began to occur by 15 min after a single CNO injection here, which is not consistent with the >60 min required to detect initial effects attributable to clozapine. Thus, selectively increasing activity of GABAergic neurons in the dorsal hindbrain regulates whole animal blood glucose.

Much of the work suggesting that *activation* of vagal drive releases glucagon relies on indirect measures of parasympathetic activity, specifically the release of pancreatic polypeptides^50^, although optogenetic activation of a subset of inhibitory, Glut2-expressing NTS neurons paradoxically increased vagus nerve activity and glucagon release in one study^36^, where the authors attributed the increase in vagal activity to the existence of a disinhibitory circuit^56^ in the DMV. On the other hand, electrical stimulation of the DMV failed to alter blood glucose levels in rats^19^. Direct measures of vagus nerve output can be contaminated by afferent activity, since the vagus nerve includes bidirectional vagal information that is dominated by afferent signaling. In one study, vagal afferent stimulation, which would be expected to activate most GABA neurons in the NTS, tended to increase blood glucose concentration, while vagal efferent stimulation decreased blood glucose concentration^67^. DMV neurons can be activated by antagonizing GABA_A_ receptors^44^, and separate populations of pancreas-projecting DMV neurons independently control endocrine versus exocrine secretions^68^. Here, hM3Dq-dependent activation of GABAergic hindbrain neurons caused an increase in GABA release in the DMV, leading to a decrease in the activity of most DMV motor neurons *in vitro*. The activation of hindbrain GABA neurons *in vivo* results in an elevation in blood glucose concentration. Therefore, we suggest that inhibition, but not excitation, of vagal motor neurons promotes the release of glucagon in the absence of a counter-regulatory release of insulin, at least at a time point corresponding to peak glucose elevation. Notably, activity changes in other cell types (e.g., glutamatergic NTS neurons^42^) could preferentially increase excitability of DMV motorneurons that regulate other metabolic regulatory systems, and such circuit-specific effects could account for the lack of significant effect on blood insulin concentration following the CNO-induced increase in GABA neuron activity. In this scenario, a glutamate-driven increase in DMV neuron activity might result in increased insulin release, which would be consistent with the hypoglycemia reported after vagal efferent stimulation^67^. Further dissection of cell- and circuit-specific activity in the dorsal vagal complex should reveal additional insight into hindbrain mechanisms that regulate metabolism.

The release of glucagon works to increase blood glucose concentration and drive hepatic production of PEPCK1, and decreased vagal drive to the liver could also contribute to increased hepatic gluconeogenesis. In the context of disease, chronic hyperglycemia elevates inhibitory, GABA_A_ receptor-mediated signaling in vagal motor neurons^48,49^, and this chronically elevated GABAergic inhibition of parasympathetic output may play a role in reinforcing the hyperglycemia and hyperglucagonemia evident in diabetes^69–71^ and/or the loss of counter-regulatory responses to hypoglycemia^72,73^. Although much work must be done to fully appreciate the hindbrain’s role in metabolic disorders like diabetes, increased GABAergic signaling within the DVC, if sustained, would thus be expected to contribute to maintaining diabetic hyperglycemia and inhibiting counter-regulatory responses to glucose changes; it could also contribute to reinstatement of hyperglycemia during brief periods of restored glycemic control. Elevated activity of brainstem inhibitory circuits therefore promotes hyperglycemia. The diabetes-related upregulation of central vagal GABA signaling reported previously^45,48,49^ may reinforce hyperglycemia.

Blood glucose concentration can be bidirectionally modulated by manipulation of central hypothalamic components of the glucose regulatory system^6^. However, we detected no change in overall blood glucose concentration after DREADDs-induced inhibition of hindbrain GABAergic neurons. Using electrophysiological recordings *in vitro*, we confirmed that hM4Di-expressing neurons were significantly hyperpolarized after CNO application and that inhibitory neurotransmission to DMV neurons was significantly reduced. These changes in neurotransmission, however, were not enough to significantly increase DMV activity *in vitro* suggesting that, at least in naïve mice, dampening of ongoing GABAergic signaling does little to alter overall DMV activity and subsequent parasympathetic regulation of glucose metabolism. Under normal conditions in the slice preparation, GABA release is relatively low, and slowing action potentials in hindbrain GABA neurons via hM4Di activity may only modestly affect DMV neuron activity, if at all, especially since DMV motor neurons have intrinsic currents that allow them to fire regularly in the absence of any neurotransmitter influence^74^. Conversely, a significant proportion of GABA_A_ receptors are normally unoccupied in DMV neurons^43,45,75^, so increasing GABA release via hM3Dq activation seems more likely to inhibit action potential firing in DMV neurons due to increased GABA_A_ receptor occupancy. Therefore, unlike hypothalamic circuitry, the activity of GABAergic neurons in the DVC regulates blood glucose concentration in a mainly unidirectional fashion: They are directly implicated in elevating blood glucose levels, but likely serve a more nuanced role (if any) in mediating decreases in blood glucose concentration. Consequently, activity in these neurons may serve as an evolutionary failsafe against hypoglycemia. Paradoxically, it could also contribute to sustained hyperglycemia in diabetes, where results from mouse models indicate that GABA sensitivity in DMV neurons is chronically increased^45,48,49^.

The DVC is a key autonomic regulatory region, receiving both peripheral afferent and descending hypothalamic and other central neural input. We and others have found that GABAergic neurons in this region are intrinsically sensitive to glucose in a glucokinase-dependent fashion^35,36^ and send significant, convergent projections to DMV motor neurons^42,44^. As the final, central nexus for parasympathetic output, DMV neurons help orchestrate signaling in peripheral organs in order to generate complex visceral responses, including those necessary to control energy homeostasis. Findings here directly link the activity of GABAergic neurons in the dorsal hindbrain to systemic glucose metabolism. These results further our understanding of how parasympathetic output influences energy homeostasis and support continued investigation of the role played by GABAergic hindbrain neurons in the parasympathetic regulation of glucose metabolism in normal and diabetic states.

## Materials And Methods

### Animals

All experiments were performed on Vgat-ires-Cre knock-in mice (i.e., vGAT-Cre mice; Slc32a1tm2(cre)lowl/J; 016962; The Jackson Laboratory, Bar Harbor, ME). Mice were housed and cared for in the University of Kentucky Division of Laboratory Animal Resources facilities according to protocols approved by the University of Kentucky Animal Care and Use Committee.

### Stereotaxic Viral Injections

At five weeks of age, mice were injected with floxed viral constructs (4 × 10^12^–2.5 × 10^13^ virus molecules/mL; 250–500 nL) into the DVC under stereotaxic control (coordinates from bregma: AP = 7.0 mm, ML = 1.0 mm, DV = 3.7 mm). Constructs, pAAV8-hSyn-DIO-hM3D(Gq)-mCherry (Addgene plasmid # 44631) or pAAV8-hSyn-DIO-hM4D(Gi)-mCherry (Addgene plasmid # 44632), were a gift from Dr. Bryan Roth and received from Addgene (Cambridge, MA). Mice were allowed to recover for one week following surgery and then acclimated to handling for 3 weeks before the start of *in vivo* studies.

### Electrophysiology

At least four weeks after viral injections, slices were prepared as previously reported using coronal brainstem slices in aCSF of a composition matching previous reports^35,48^. Coronal slices (300 µm) containing the DVC were prepared and transferred to a holding chamber oxygenated (95%O_2_/5%CO_2_) at 32–34 °C. On cell or whole-cell patch-clamp recordings were performed using glass pipettes (2–5 MΩ). Internal recording solution composition matched previous reports^35,48^. Action potentials were recorded at resting membrane potential in current-clamp mode. DMV cells were voltage-clamped at a holding potential of 0 mV to record IPSCs. Kynurenic acid (KYN; 1 mM) was added to perfusate for recordings in the DMV to block synaptic currents mediated by ionotropic glutamate receptors and isolate GABAergic currents; bicuculline methiodide (BIC; 30 µM) was used to block GABA_A_ receptor-mediated currents.

All recordings were low-pass filtered at 3 kHz and acquired digitally at 20 kHz using pClamp acquisition software (Axon Clampfit; Molecular Devices, San Jose, CA). Mini-analysis (Synaptosoft, Decatur, GA) was used to measure action potential firing and IPSC frequency and amplitude.

### Single-cell RT-PCR

Single-cell RT-PCR was performed as previously reported^35^. The oligonucleotides used for PCR targeted β-actin (verifying the presence of constituent mRNA) and GAD67 (identifying GABAergic cells). Cells were required to be positive for β-actin expression in order to be considered for analysis. Two control samples were run: One contained reagents only (NTC); for the other, a pipette was filled with internal solution, placed on the surface of the slice for five minutes and then the solution was expelled into an RNase-free PCR tube exactly as when cell contents are collected.

### *In vivo* Glucose Assessments

Four weeks after viral delivery, mice were randomly selected to receive either vehicle (0.9% saline + 0.5% DMSO) or CNO (1 mg/kg). Mice were fasted for two hours before getting an i.p. injection of either vehicle, CNO, or glucose (0.26 g/kg; to match the rise after CNO administration). Animals remained fasted and blood glucose was measured (tail vein lance; OneTouch Ultra) for 5 hrs. One cohort was pre-treated with MSA (1 mg/kg; i.p.) 15 min prior to CNO or vehicle injection. Each animal received the opposite treatment 3–4 days later to provide a counter-balanced design. Data are expressed as the change in blood glucose measured immediately prior to injection.

### Immunohistochemistry and Imaging

All injection sites were confirmed after experiment termination using endogenous mCherry expression. To confirm electrophysiological recording locations, slices were fixed and biocytin (0.1%) filled neurons were stained for avidin-AMCA (1:400; Vector Laboratories, Burlingame, CA) after recording as previously described^35,76,77^. Imaging was done with an Olympus BX40 microscope, and images were captured with a Spot RT camera (Diagnostic Instruments, Burroughs, MI) using filters for the two fluorescent dyes (Fig. 1).

### Western Blots

Frozen tissues were homogenized in RIPA buffer with Halt Protease Inhibitor Cocktail (Thermo Fisher 78430) and Halt Phosphatase Inhibitor (Thermo Fisher 78420). Concentration was measured using DC Protein Assay (Biorad 5000116). Samples were boiled with SDS sample buffer for loading. Protein homogenate (30 µg) in SDS sample buffer was subjected to SDS-PAGE and transferred onto PVDF membranes. Membranes were blocked with 5% milk in TBS-T (TBS, 0.1% TWEEN 20). Membranes were incubated overnight at 4 °C with primary antibodies (1:1,000) from Cell Signaling phospho-LDHA (#8176), LDHA (#2012), AKT (#9272), phospho-AKT S473 (#4058). Membranes were washed in TBS-T and incubated for 1 h at room temperature with the secondary antibody (Invitrogen A21109). Fluorescent secondary antibody was detected on Licor Odyssey and analysis of band intensity was performed in ImageJ.

### Data Analysis

Recordings were analyzed using Clampfit 10.2 (Molecular Devices) and MiniAnalysis 6.0.7 software (Synaptosoft). For analysis of postsynaptic currents and action potential firing, two minutes of continuous recording under each condition was used. Changes in action potential firing in DMV neurons from baseline of ≥15% were considered significant. Group mean ± standard error of the mean (SEM) is reported. A repeated-measures ANOVA with Tukey’s post hoc was used to determine if CNO injection significantly changed blood glucose. For all other data, a paired or unpaired, two-tailed Student’s *t-*test was used to determine statistical significance (Graphpad Prism; La Jolla, CA). Unless otherwise stated, statistical significance for all measures was set at *p* ≤ 0.05.

## Supplementary information

Supplemental figures

## Data Availability

The authors will make materials, data and associated protocols promptly available to readers without undue qualifications in material transfer agreements.
