# Supplementary material for: A hindbrain inhibitory microcircuit mediates vagally-coordinated glucose regulation
Source: Sci Rep. 2019 Feb 25;9:2722. doi: 10.1038/s41598-019-39490-x (PMC6389891; doi:10.1038/s41598-019-39490-x)
Supplement: Supplementary file 1 — Supplemental figures [file 41598_2019_39490_MOESM1_ESM.docx]

**Supplemental Information**

**A hindbrain inhibitory microcircuit mediates vagally-coordinated glucose regulation**

Carie R. Boychuk^1^, Katalin Cs. Smith^1^, Laura E. Peterson^1^, Jeffery A. Boychuk^1^, Corwin R. Butler^1^, Isabel D. Derera^1^, John J. McCarthy^1^ and Bret N. Smith^1,2^

^1^Department of Physiology, College of Medicine, University of Kentucky; ^2^Department of Neuroscience, College of Medicine, University of Kentucky

**Supplemental Figure S1**. **There was no effect of CNO in mice that did not express the hM3Dq construct in the DVC.** In mice that received either saline injection in the NTS or in which the pAAV8-hSyn-DIO-hM3Dq-mCherry injection was off target and did not result in mCherry expression in the DVC, CNO treatment was without effect on blood glucose concentration. In all animals, however, there was a significant effect of time, reflecting the lowering of blood glucose in fasted mice. * indicates significant difference from time 0, but no difference between groups.

**Supplemental Figure S2.** **Peripheral muscarinic receptor blockade increased blood glucose concentration.**  MSA (1 mg/kg; i.p.) or saline was injected 15 min prior to vehicle or CNO treatment. Blood glucose concentration was significantly increased by MSA (p=0.005).

**Supplemental Figure S3.** **Effects of CNO and glucose treatment on expression of LDHA and pLDHA.** a) CNO administration significantly decreased total LDHA expression compared to either vehicle, saline, or glucose. b) CNO administration significantly decreased total pLDHA expression compared to glucose. * indicates significant difference from CNO.

**Supplemental Figure S4.**  **Western blots showing pAKT, AKT and pLDHA, LDHA protein expression in gastrocnemius muscle.** The full blots from data shown in Figure 3e are shown. a. Full Western blots showing LDHA and pLDHA expression in mice that received saline vehicle, CNO, or glucose injections. b. Full blots showing AKT and pAKT expression in the three groups.

Fig S1


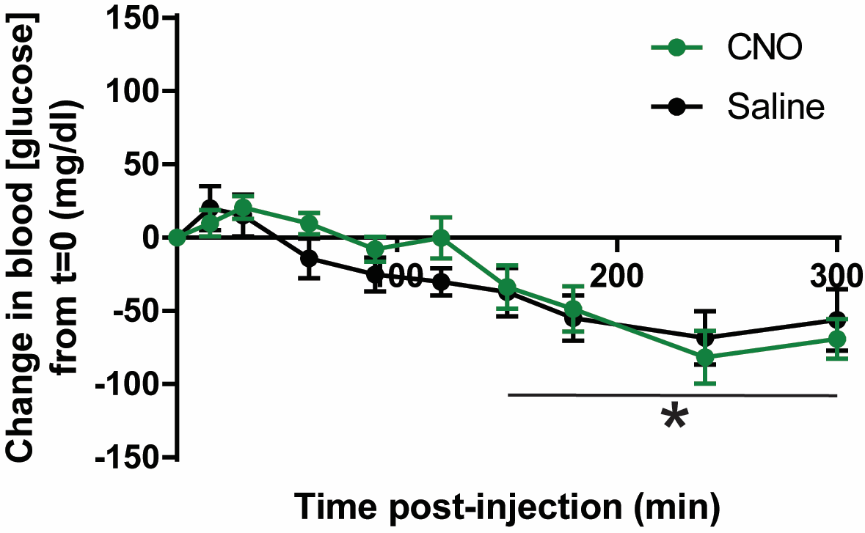


Fig S2





Fig S3


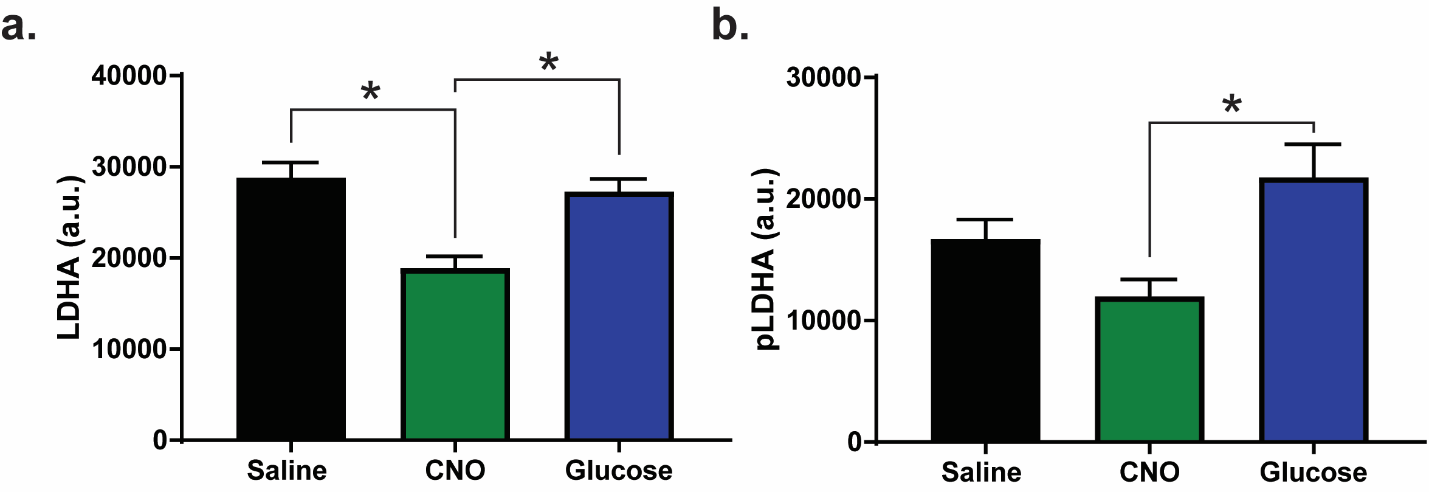


Fig S4
